# Supplementary material for: A Nutraceutical Formulation Containing Brown Algae Reduces Hepatic Lipid Accumulation by Modulating Lipid Metabolism and Inflammation in Experimental Models of NAFLD and NASH
Source: Mar Drugs. 2022 Sep 8;20(9):572. doi: 10.3390/md20090572 (PMC9501409; doi:10.3390/md20090572)

Materials and methods

The most abundant components of the commercial nutraceutical product (Gdue©) were identified by direct infusion (DI) – high resolution mass spectrometry (HRMS). Briefly, two different 100 mg aliquots of commercial product were extracted with ultrapure water and methanol, respectively, sonicated for 15 minutes and centrifuged at 15000 x g for 10 minutes. Supernatants were further diluted 1:10 with a 50:50 water/methanol + 0.1% formic acid solution and directly analyzed by DI-HRMS.

Analysis was performed by a Q-Exactive hybrid quadrupole-Orbitrap mass spectrometer (Thermo Fisher Scientific,Waltham, MA, USA), in both positive and negative electrospray mode. The capillary voltage was 4.0 kV and -2.8kV in positive and negative mode respectively, the capillary temperature was 320 °C, auxiliary gas and sheath gas were nitrogen at 20 and 10 a.u., respectively. Resolution was set to 70000 in MS mode and 35000 in MS/MS mode, while AGC target was $1x10^6$, max injection time 250 ms and scan-range 100-1500 Da. MS/MS experiments in HCD mode were performed by setting an isolation window of 2 m/z and by manually optimizing the fragmentation energy for each parent ion of interest.

Calibration was performed with Pierce ESI Positive Ion Calibration Solution (Thermo Fisher Scientific, Waltham, MA, USA) and Pierce ESI Negative Ion Calibration Solution (Thermo Fisher Scientific, Waltham, MA, USA). The MS data were analyzed with the Xcalibur 4.0 software (Thermo Fisher Scientific,Waltham, MA, USA).

Results

The DI-HRMS analysis of aqueous and methanolic extracts, although limited to the most abundant molecular species, confirmed that the commercial nutraceutical product contains polysaccharides and polyphenols, and also highlighted the presence of sulfolipids, i.e. different sulfoquinovosyl-diacyl-glycerol (SQDG) analogues.

The presence of alditol-based oligo- or polysaccharides was confirmed by the ion signals at *m/z* 205.0681, 367.1206, 529.1742, 691.2261, 853.2780 and 1015.3286 obtained by the analysis of the aqueous extract in positive ion mode (Figure S1). Indeed, the reported ion signals showing a mass difference of 162.0528 Da are compatible with sodium adduct fragments of O-linked oligosaccharides or O-linked oligosaccharide containing molecules, obtained by in-source fragmentation. [10.1093/glycob/cwg089; 10.1016/j.carbpol.2003.11.001].

Sulfolipids were identified in the methanolic extract of the nutraceutical product by DI-HRMS in negative ionization mode (Figure S2), and further characterized by MS/MS (Figure S3) to clarify the fatty acid composition. The main identified SQDG analogues were detected as deprotonated ions: SQDG C16:0/C14:0 (*m/z* 765.4846, MS/MS in Figure S3.a), SQDG C18:1/C14:0 (*m/z* 791.5004, MS/MS in Figure S3.b), SQDG C18:0/C14:0 and SQDG C16:0/C16:0 (*m/z* 793.5156, MS/MS in Figure S3.c), SQDG (18:1/16:0) (*m/z* 819.5219, MS/MS in Figure S3.d), SQDG (24:1/14:0) (*m/z* 875.5941, MS/MS in Figure S3.e), and SQDG (24:1/16:0) (*m/z* 903.6258, MS/MS in Figure S3.f), [10.1515/znc-2011-3-409.]. In Figure S2 it is also possible to observe signals in the range 500-600 m/z, due to the in-source fragmentation of the identified SQDGs, as already reported for other lipid classes [10.1002/jms.3439.]

Some polyphenols were also identified in the aqueous extract by DI-HRMS in positive ionization mode, at quite low signal intensities. In particular, nobiletin (Figure S4.a) and niranthin (Figure S4.b) were identified by comparison of the MS/MS spectra with the online MS/MS database mzCloud (https://www.mzcloud.org). The comprehensive characterization of the polyphenolic composition of the nutraceutical product is still in progress and it is beyond the purpose of the present investigation.

Figure S1: DI-HRMS spectrum, in electrospray positive ionization mode, of the nutraceutical product aqueous extract.

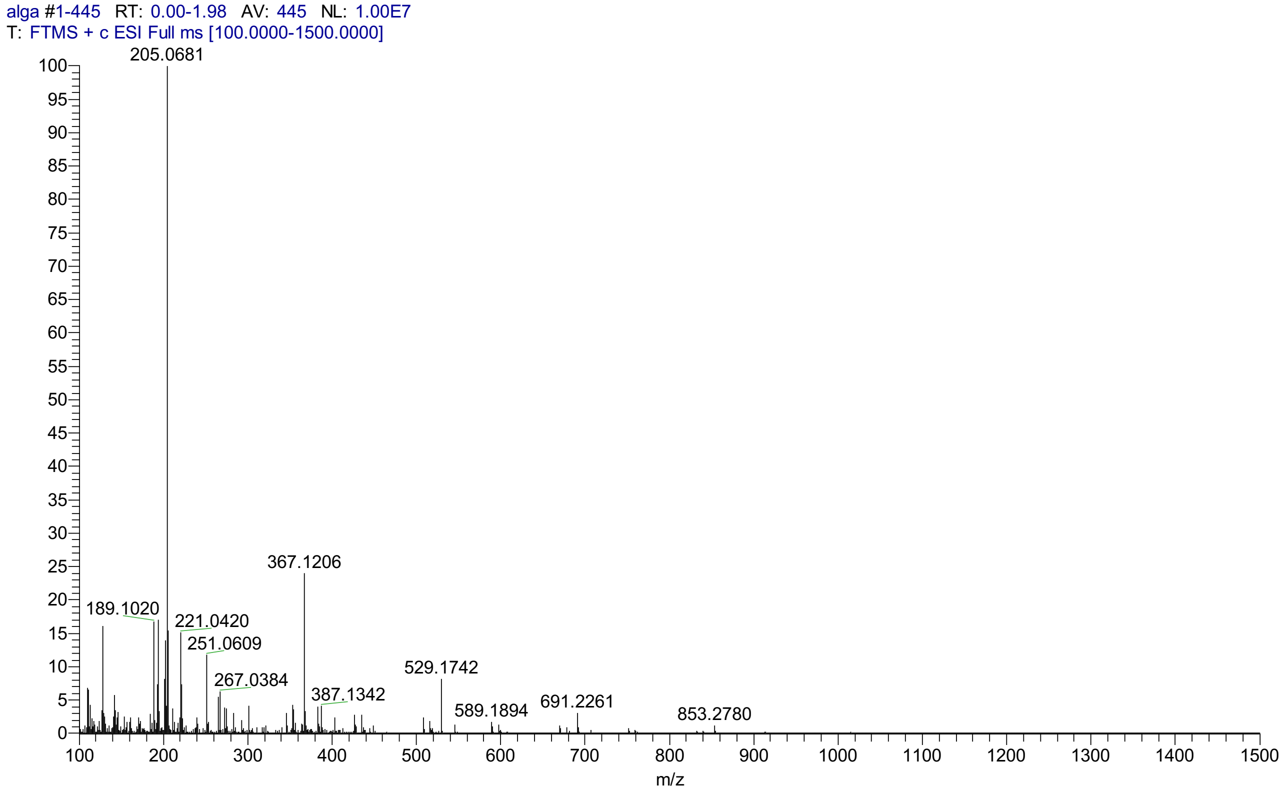

Figure S2: DI-HRMS spectrum (zoom in the range 500-1100 *m/z*), in electrospray negative ionization mode, of the nutraceutical product methanolic extract.

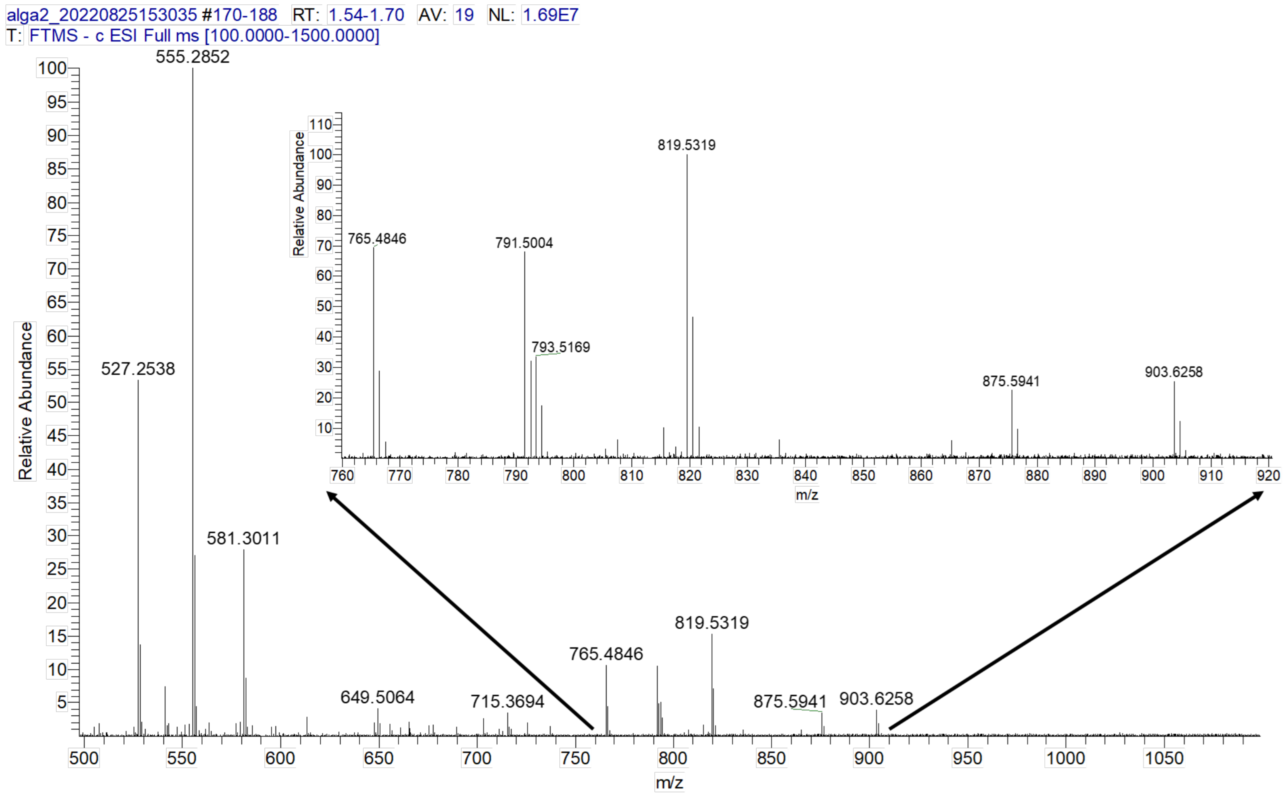

Figure S3: MS/MS spectra of detected SQDG analogues. Data were acquired by setting a normalized collision energy (NCE) of 40 in HCD mode.

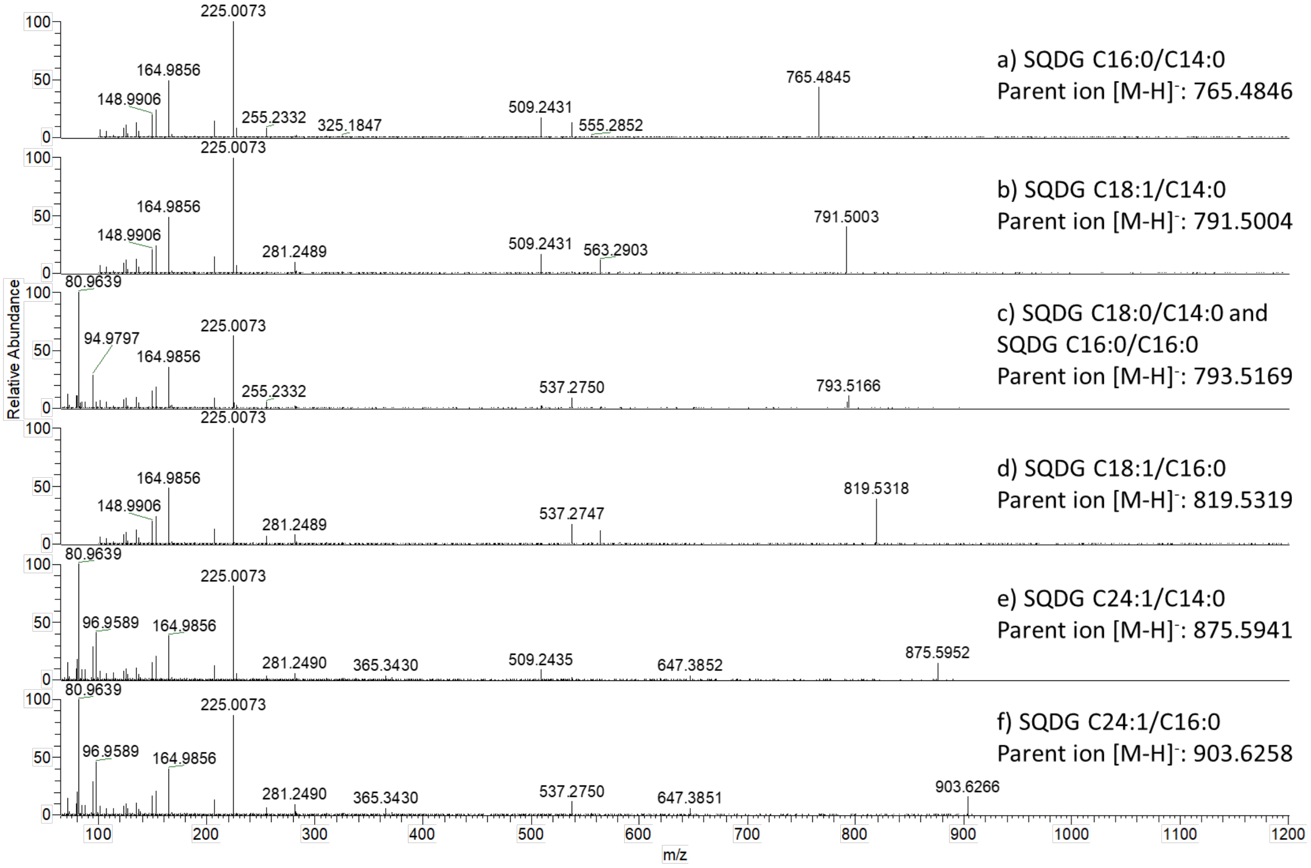

Figure S4: MS/MS spectra obtained for a) nobiletin and b) niranthin. Data were acquired by setting a normalized collision energy (NCE) of 30 in HCD mode.

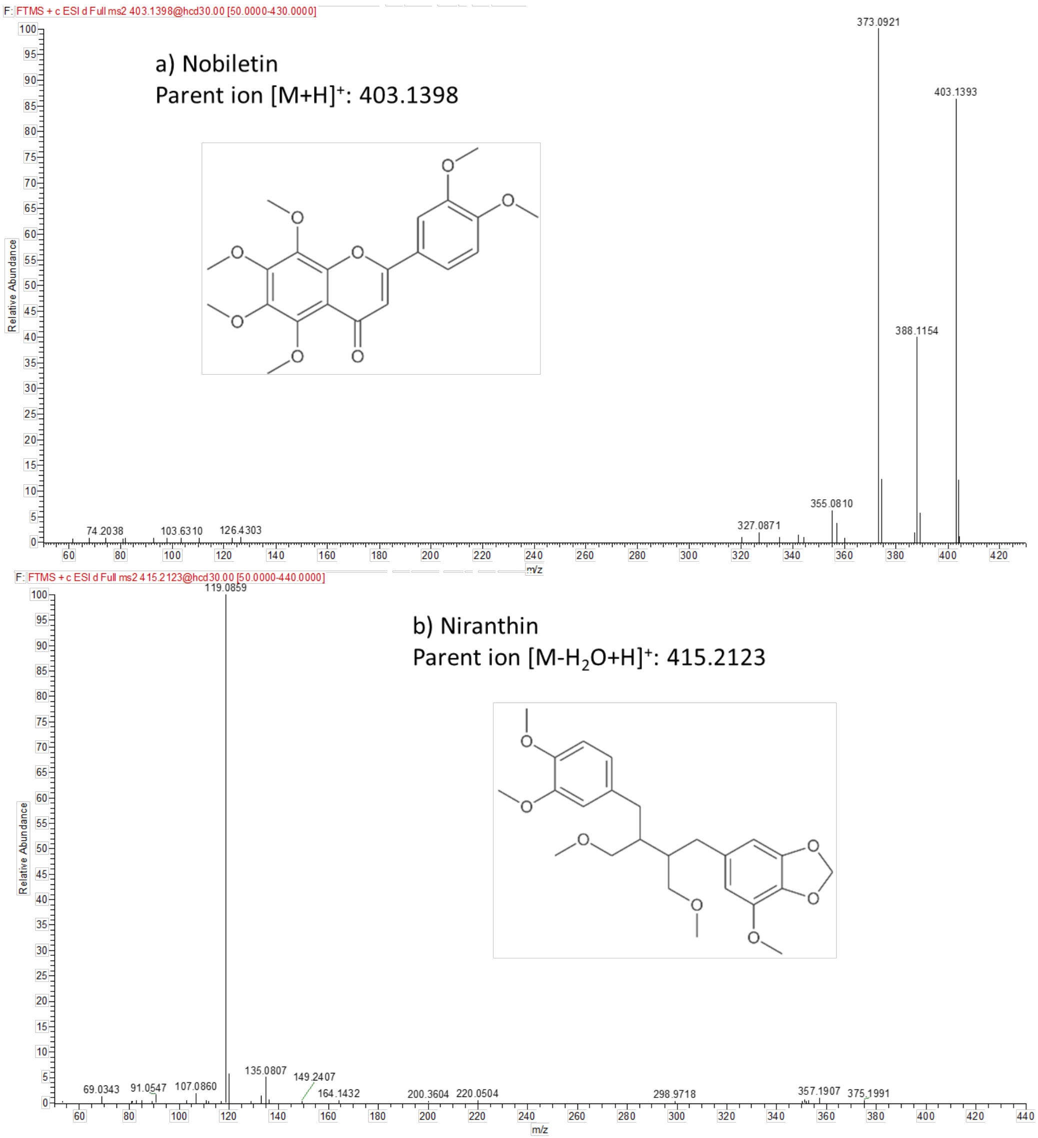

Supplement: Supplementary file 1 [file marinedrugs-20-00572-s001.zip › marinedrugs-1880276-supplementary.pdf]
